# Temporal Changes in Glutaredoxin 1 and Protein S-Glutathionylation in Allergic Airway Inflammation

**DOI:** 10.1371/journal.pone.0122986

**Published:** 2015-04-13

**Authors:** Kanako Maki, Katsura Nagai, Masaru Suzuki, Takashi Inomata, Takayuki Yoshida, Masaharu Nishimura

**Affiliations:** First Department of Medicine, Hokkaido University School of Medicine, Sapporo, Japan; The Hospital for Sick Children and The University of Toronto, CANADA

## Abstract

**Introduction:**

Asthma is a chronic inflammatory disorder of the airways, involving oxidative stress. Upon oxidative stress, glutathione covalently binds to protein thiols to protect them against irreversible oxidation. This posttranslational modification, known as protein S-glutathionylation, can be reversed by glutaredoxin 1 (Glrx1) under physiological condition. Glrx1 is known to increase in the lung tissues of a murine model of allergic airway inflammation. However, the temporal relationship between levels of Glrx1, protein S-glutathionylation, and glutathione in the lungs with allergic airway inflammation is not clearly understood.

**Methods:**

BALB/c mice received 3 aerosol challenges with ovalbumin (OVA) following sensitization to OVA. They were sacrificed at 6, 24, 48, or 72 h, or 8 days (5 mice per group), and the levels of Glrx1, protein S-glutathionylation, glutathione, and 25 cytokines/chemokines were evaluated in bronchoalveolar lavage fluid (BALF) and/or lung tissue.

**Results:**

Levels of Glrx1 in BALF were significantly elevated in the OVA 6 h (final challenge) group compared to those in the control, with concurrent increases in protein S-glutathionylation levels in the lungs, as well as total glutathione (reduced and oxidized) and oxidized glutathione in BALF. Protein S-glutathionylation levels were attenuated at 24 h, with significant increases in Glrx1 levels in lung tissues at 48 and 72 h. Glrx1 in alveolar macrophages was induced after 6 h. Glrx1 levels concomitantly increased with Th2/NF-κB-related cytokines and chemokines in BALF.

**Conclusions:**

The temporal relationships of Glrx1 with protein S-glutathionylation, glutathione, and cytokines/chemokines were observed as dynamic changes in lungs with allergic airway inflammation, suggesting that Glrx1 and protein–SSG redox status may play important roles in the development of allergic airway inflammation.

## Introduction

Asthma is a chronic inflammatory disorder of the airways caused by exposure to various allergens and chemical irritants in susceptible subjects. Oxidative stress is thought to play a pathophysiological role in the disease by causing damage to airway epithelial cells, leading to airway hyperresponsiveness and airflow limitation.

The tripeptide glutathione (GSH; l-γ-glutamyl-l-cysteinyl-glycine), which is highly abundant in cells and lung epithelial lining fluid, acts as an antioxidant and plays a major role in maintaining overall redox homeostasis. Agents that cause oxidative stress are known to decrease the ratio of reduced GSH to oxidized glutathione (glutathione disulfide or GSSG). Elevated levels of GSSG can be considered a marker of oxidative stress, whereas increased total or reduced GSH levels can be regarded as an adaptive response to increased oxidative burden in the lungs [1–3]. As an antioxidant, GSH might conjugate with reactive cysteines in proteins under conditions of oxidative stress. This posttranslational modification is termed variously as protein S-glutathionylation (protein—SSG), S-glutathiolation, or protein mixed disulfides. Protein—SSG modifications change the structure and function of proteins in a reversible and tightly regulated manner. Protein—SSG disrupts the function of nuclear factor κB (NF-κB) [4, 5], which is an important transcription factor that regulates allergic airway inflammation [6–8].

Mammalian glutaredoxin enzymes are members of the thioredoxin family of thiol transferases. Glutaredoxin specifically catalyzes de-glutathionylation under physiological conditions, which restores the reduced sulfhydryl groups of the cysteines of proteins [3, 9, 10]. The mRNA and protein expression, as well as activity, of glutaredoxin 1 (Glrx1) were found to increase in lung tissues from mice with ovalbumin (OVA)-induced allergic airway inflammation [11]. However, the temporal relationship between levels of Glrx1 and protein—SSG in the lungs of a murine model after OVA challenge remains unclear. Furthermore, although the kinetics of helper T cell type 2 (Th2) cytokines in BALF after OVA challenge have been reported [12], the temporal relationship between cytokines and Glrx1 has not been investigated.

The goal of the present study was to investigate the temporal relationships of Glrx1 with protein—SSG, glutathione, and Th2/NF-κB-related cytokines/chemokines using a well-characterized model of OVA-induced allergic airway inflammation. Understanding such temporal relationships is important to clarify the cascade of various molecules during the course of an asthma attack. This might provide clues to break the vicious cycle.

## Materials and Methods

### Study animals

All animal experiments were approved by the Ethics Committee for Animal Research at Hokkaido University (11–0084). Female BALB/c mice (aged 6–7 weeks) were purchased from CLEA Japan (Tokyo, Japan). All mice were housed in plastic chambers with free access to food and water.

### Experimental design

For induction of experimental allergic lung disease, sensitization and challenges were performed according to a previously published method [13] with some modifications. Briefly, mice were immunized intraperitoneally with 200 μL phosphate-buffered saline (PBS) containing 50 μg OVA (Grade V; Sigma-Aldrich, St. Louis, MO) plus 4.0 mg aluminum hydroxide adjuvant (Imject Alum; Thermo Scientific, Rockford, IL) on days 0 and 7. Mice (5 per group) were challenged with inhaled allergen (2.5% OVA in PBS) for 20 min or with PBS alone (control group) on days 21, 22, and 23. For this procedure, mice were placed in a plastic chamber (40 × 25 × 13 cm) and administered the OVA solution via an ultrasonic nebulizer (NE-U17; Omron Healthcare, Kyoto, Japan). The mice were euthanized with an overdose of ketamine and xylazine for the collection of BALF and lung tissues at 6, 24, 48, or 72 h, or 8 days after the third inhalation challenge (day 23).

### Bronchoalveolar lavage, cell count analysis, and cytokine/chemokine profiling

Mice were anesthetized, and a 22-gauge cannula was inserted into the trachea of each mouse. Cells were collected by washing the airway lumen 3 times with 0.6 mL of saline. The supernatants were stored at −80°C until analysis. For differential cell count, cells were prepared using cytospin and stained with Diff-Quik (International Reagent Corp., Hyogo, Japan). Cytokine and chemokine concentrations in BALF were measured using a MILLIPLEX Mouse Cytokine/Chemokine kit (Millipore, Billerica, MA), which included eotaxin-1, G-CSF, GM-CSF, IFN-γ, IL-1α, IL-1β, IL-3, IL-4, IL-5, IL-6, IL-9, IL-10, IL-12 (p40), IL-12 (p70), IL-13, IP-10, KC, MCP-1, MIP-1α, MIP-2, RANTES, TNF-α, TGF-β1, TGF-β2, and TGF-β3.

### Glrx1 expression in lung tissues and BALF

Lung homogenates and BALF were resolved by sodium dodecyl sulfate polyacrylamide gel electrophoresis and blotted onto a polyvinylidene fluoride membrane (Bio-Rad Laboratories, Hercules, CA). The membrane was blocked at room temperature for 1 h in 2% skim milk powder in 25 mM Tris (pH 7.4) and 0.15 M NaCl containing 0.1% Tween 20. The membrane was incubated overnight at 4°C with primary antibody against Glrx1 (1:1000; R&D Systems, Minneapolis, MN). After 3 washes with Tris-buffered saline (TBS) with Tween, the peroxidase-conjugated secondary antibody (1:4000; R&D Systems) was incubated for 1 h at room temperature. The conjugated peroxidase was detected by chemiluminescence using the Immobilon Western Chemiluminescent HRP Substrate (Millipore) followed by scanning with a luminescent image analyzer, LAS-4000 mini-series (Fujifilm, Tokyo, Japan). In lung homogenates, on each blot, the detected Glrx1 band intensity was divided by the actin band intensity as a loading control and then divided by the value of the representative standard (Glrx1/actin), which was used on every blot. In BALF, on each blot, the detected Glrx1 band intensity was divided by that of a representative standard sample at 6 h after the last challenge. Glrx1 content is expressed in arbitrary units.

### Immunohistochemical localization of Glrx1

For *in situ* detection of Glrx1 levels, immunohistochemical staining was employed, as described previously [14], with the avidin-biotin-peroxidase complex method followed by hematoxylin counterstaining. The appearance of a dark brown color indicated the presence of Glrx1 in lung tissues. In brief, formalin-fixed, paraffin-embedded lung sections (4-μm-thick) were deparaffinized and rehydrated by passage through a xylene and graded alcohol series. Endogenous peroxidase activity was quenched by exposure to 3% H_2_O_2_ in methanol for 30 min. Nonspecific binding of antibodies to the tissue sections was blocked by incubation with goat serum (Vector Laboratories, Burlingame, CA) for 1 h. Tissue sections were incubated with Glrx1 rabbit polyclonal antibody (1:100; Santa Cruz Biotechnology, Santa Cruz, CA) overnight at 4°C. As a negative control, normal rabbit IgG (Vector Laboratories) was used instead of Glrx1 rabbit polyclonal antibody. After washing with PBS, tissue sections were incubated with secondary antibody (Vector Laboratories) for 1 h. 3,3ʹ-Diaminobenzidine (Vector Laboratories) was used as the peroxidase substrate. In each instance, sections from different groups were processed together, with equal time for color development.

### Fluorescent immunostaining for Glrx1 expression in alveolar macrophages

For the analysis of cells in BALF, BALF was centrifuged onto glass slides, fixed with 4% paraformaldehyde in PBS, and stored at −80°C. Slides were then blocked for 1 h with TBS containing 2.5% bovine serum albumin, 2.5% goat serum IgG, and 0.5% Tween. Slides were then incubated with Glrx1 primary antibody (1:200; Santa Cruz Biotechnology) and anti-F4/80 antibody (1:100; Bio-Rad Laboratories) for alveolar macrophages in blocking buffer at 4°C overnight. Slides were washed with TBS and incubated for 1 h with fluorophore-conjugated secondary antibodies (1:1000; Invitrogen) in blocking buffer. Slides were washed with TBS, and nuclei were stained with 4ʹ,6-diamidino-2-phenylindole (DAPI) (Vector Laboratories). Slides were analyzed by confocal microscopy using an Olympus Fluoview 300 microscope (Olympus, Center Valley, PA).

### *In situ* detection of S-glutathionylated proteins in lung tissues following Glrx1-catalyzed cysteine derivatization

*In situ* analysis of protein—SSG in lung tissues was performed as described previously [15, 16] with slight modifications. In brief, formalin-fixed, paraffin-embedded lung sections were deparaffinized and rehydrated by passing them through a xylene and graded alcohol series. Free thiol groups were blocked with a buffer containing 25 mM HEPES (pH 7.4), 0.1 mM EDTA (pH 8.0), 0.01 mM neocuproine (Sigma-Aldrich), 40 mM *N*-ethylmaleimide (Sigma-Aldrich), and 1% Triton X (Sigma-Aldrich). After the lung sections were washed with PBS, S-glutathionylated cysteine groups were reduced by incubation with 13.5 mg/mL human Glrx1 (Lab Frontiers, Seoul, Korea), 35 mg/mL glutathione reductase (Roche Diagnostics GmbH, Mannheim, Germany), 1 mM GSH (Sigma-Aldrich), 1 mM β-NADPH (Sigma-Aldrich), 18 mmol EDTA, and 137 mM Tris·HCl (pH 8.0). After washing with PBS, the reduced cysteine was labeled with 1 mM N-(3-maleimidylpropionyl) biocytin (MPB; Invitrogen, Carlsbad, CA). Excess MPB was removed, and the tissue samples were incubated with streptavidin-conjugated Alexa Fluor 568 (Invitrogen). Nuclei were stained with Sytox Green (Invitrogen). For the negative control, Glrx1 alone or Glrx1, GSH, GSSG reductase, and NADPH were omitted from the reaction mix. For the positive control, the tissue sample was incubated with 400 mol/L diamide (Sigma-Aldrich) and 1 mmol/L GSH for 10 min before application of *N*-ethylmaleimide. Slides were analyzed by confocal microscopy as previously described. Quantitative assessment of the intensity of protein—SSG reactivity in the bronchial epithelium was conducted by evaluating the mean red fluorescence intensity (protein—SSG) in each region of interest and dividing this by the mean green fluorescence intensity (DNA content) present in the same region, thereby obtaining the relative fluorescence intensity (RFI) of protein—SSG. Bronchial epithelium regions were selected based on their architectural appearance, and sections that were stained with hematoxylin and eosin were examined. The RFI for protein—SSG was analyzed from images in the jpeg format using Image J (version 1.45).

### Biochemical analysis of protein—SSG in lung tissues

protein—SSG in lung tissues was determined using a glutathione/glutathione reductase/NADPH/5,5ʹ-dithiobis-(2-nitrobenzoic acid) (DTNB) recycling assay according to procedures described by Rahman [17]. Lungs were perfused, lavaged, and homogenized in 9 mL of 5% sulfosalicylic acid per gram tissue and then centrifuged at 2500 × *g* for 10 min at 4°C. The pellet was treated with 1 mL of 1% NaBH_4_ to remove GSH from the protein—SSG, neutralized with 0.4 mL 30% metaphosphoric acid, and centrifuged at 1000 × *g* for 15 min. The supernatant in the GSH assay (as described below) was calculated as nmol/mg of lung tissue.

### Measurement of total GSH and GSSG in lung tissues and BALF

Total GSH (reduced and oxidized GSH) and oxidized GSH (glutathione disulfide: GSSG) were measured in deproteinated lung homogenates and BALF using the DTNB recycling assay as described previously [17, 18], with slight modifications. Lung tissues were homogenized in 10 mL of 5% sulfosalicylic acid (Sigma-Aldrich) per gram of tissue. After centrifugation at 2500 × *g* for 10 min at 4°C, the supernatants were diluted to 1:8 with 0.1 M potassium phosphate buffer containing 5 mM EDTA, and these solutions were used for the total GSH and GSSG assays. For the total GSH assay, 20 μL of the standard or sample was added to each well. Equal volumes of freshly prepared DTNB (Sigma-Aldrich) and glutathione reductase (Roche Diagnostics GmbH) solutions were mixed together, and 120 μL was added to each well. After 30 s, 60 μL of β-NADPH (Sigma-Aldrich) was added, and the absorbance (412 nm) was immediately read using a microplate reader. Measurements were repeated every 30 s for 2 min, and the rate of 2-nitro-5-thiobenzoic acid formation (change in absorbance per minute) was calculated. The actual total GSH concentration in the samples was then determined using linear regression. For the GSSG assay, 100 μL of the standard or sample and 2 μL of 2-vinylpyridine (Sigma-Aldrich) were added to a 1.5-mL glass tube and then mixed well. After 1 h at room temperature, 6 μL of triethanolamine (Sigma-Aldrich) was added; the final pH was targeted to be between 6 and 7. Afterwards, the derivatized samples and GSSG standards were determined by the same method used to determine total GSH. The BALF used in these assays was unaltered and examined by the same method used to evaluate lung homogenate supernatants.

### Data analysis

All data are presented as means ± SEM. Statistical analysis was performed using one-way ANOVA with Dunnett’s multiple comparison test or the Games—Howell test. Correlations were assessed using Pearson’s correlation coefficient. All tests were performed using StatView-J 5.0 (SAS Institute, Cary, NC). p < 0.05 was considered to be statistically significant.

## Results

### Time-dependent cellular infiltration in BALF

We first investigated the characteristics of BALF to assess the time course of allergic airway inflammation after OVA challenge. The total number of inflammatory cells in the BALF of all OVA-challenged mice was significantly higher than in control mice at all the time points except 6 h after the last challenge (p < 0.05). A differential cell count analysis showed a significantly higher number of eosinophils, neutrophils, and macrophages in the BALF of OVA-challenged mice (p < 0.05). Neutrophil infiltration peaked early, at 6 h, and returned to the baseline by 8 days. In contrast, eosinophil numbers in BALF did not increase until 24 h and peaked at 72 h, remaining elevated for 8 days (Table 1).

**Table 1:** Characteristics of bronchoalveolar lavage fluid (BALF) after ovalbumin (OVA) sensitization and challenges.

	control	OVA 6 h	OVA 24 h	OVA 48 h	OVA 72 h	OVA 8day
Total cells (10^4^/ml)	2.6±0.5	9.1±1.9	12.4±2.0	14.4±1.6*	17.3±2.5*	10.8±1.7*
Macrophages (10^4^/ml)	2.6±0.5	3.2±0.4	2.9±0.2	3.5±0.5	5.5±0.8*	4.8±0.7*
[%]	[99.9]	[39.2]	[25.5]	[25.2]	[32.1]	[46.0]
Neutrophils (10^4^/ml)	0.0±0.0	2.1±0.5*	1.9±0.3*	1.5±0.2*	1.0±0.1*	0.1±0.1
[%]	[0.1]	[22.3]	[16.4]	[10.9]	[6.4]	[1.1]
Eosinophils (10^4^/ml)	0.0±0.0	3.7±1.0	7.1±1.7*	9.2±1.3*	10.4±1.7*	5.1±1.1*
[%]	[0]	[37.1]	[54.7]	[62.9]	[58.9]	[46.3]
Lymphocytes (10^4^/ml)	0.0±0.0	0.1±0.1	0.4±0.3	0.1±0.0	0.4±0.1	0.8±0.2*
[%]	[0]	[1.1]	[3.1]	[0.8]	[2.3]	[6.3]

Data are presented as means ± SEM (n = 5).

*p <0.05 compared with control mice.

### Expression of Glrx1 in BALF and lung tissues

**Fig 1:**
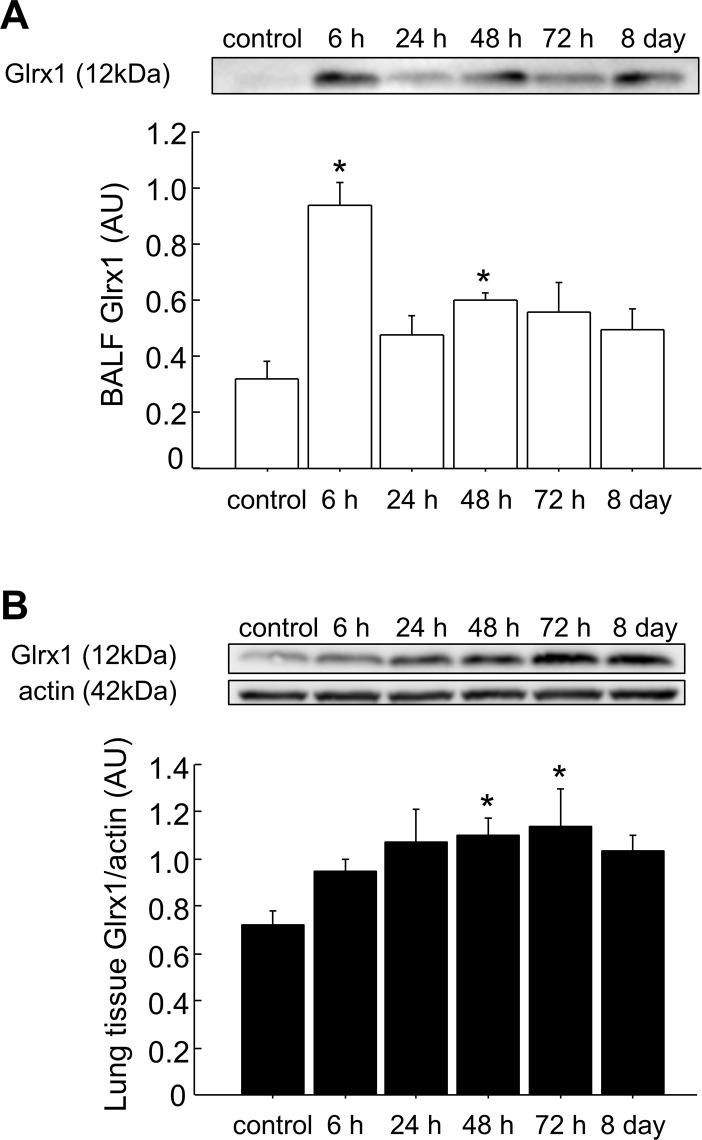
Expression of Glrx1 in the BALF and lung tissues of mice after OVA challenges. BALF (A) and lung homogenates (B) from OVA-challenged mice were analyzed by western blot analysis for Glrx1 expression at the indicated time points (6, 24, 48, and 72 h, and 8 days after the last challenge with OVA). Actin was used as a loading control for lung homogenates; data are presented as means ± SEM. *p < 0.05 was considered significant in the comparisons with control mice.

**Fig 2:**
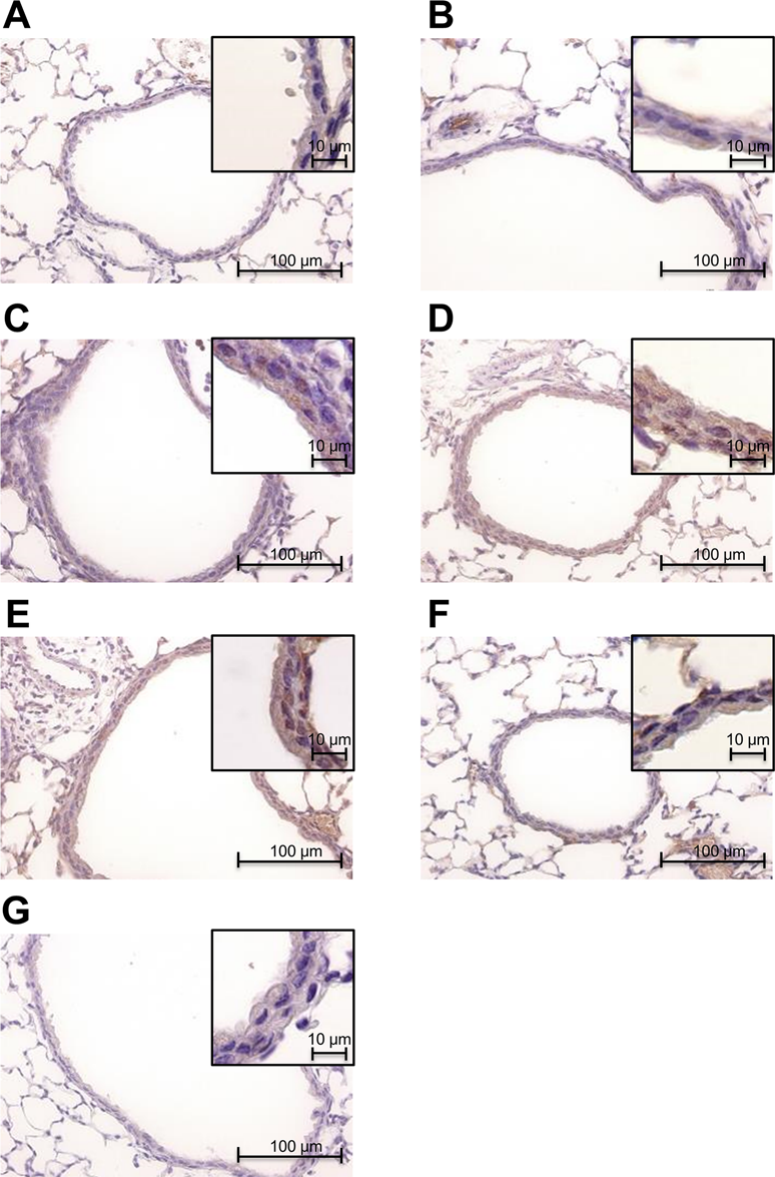
Immunohistochemical detection of Glrx1 expression in the bronchial epithelia of mice after OVA challenges. Glrx1-positive cells were identified by dark brown immunohistochemical staining. Expression of Glrx1 in murine lung tissue of PBS control (A), and 6 h (B), 24 h (C), 48 h (D), 72 h (E), and 8 days (F) after the last OVA challenge. Normal rabbit IgG was used instead of Glrx1 rabbit polyclonal antibody as a negative control (G). Magnification, ×400.

**Fig 3:**
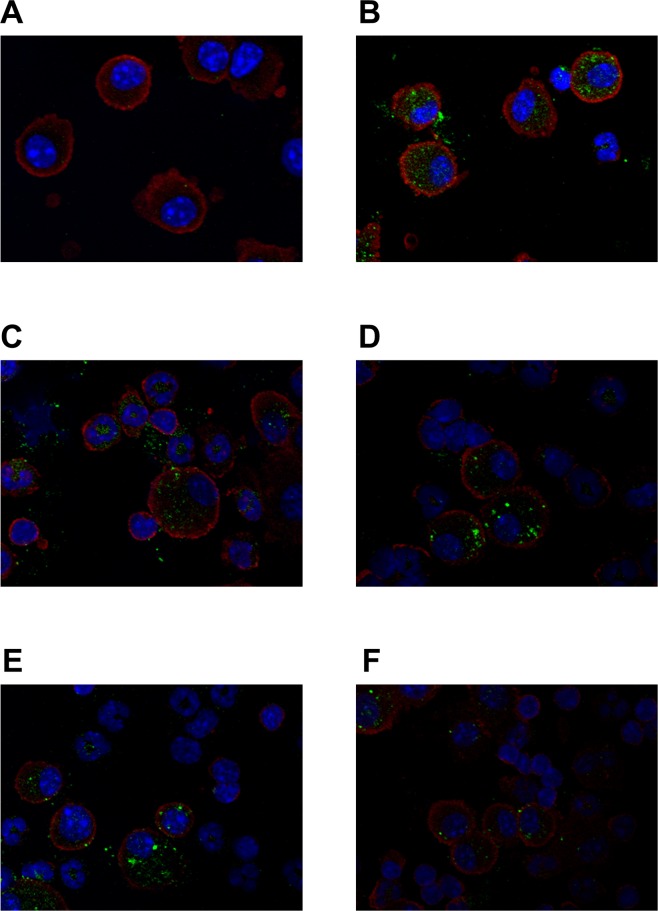
Fluorescent immunostaining detection of Glrx1 expression in alveolar macrophages of mice after OVA challenges. Representative immunofluorescence images of alveolar macrophages stained for Glrx1. Expression of Glrx1 in murine alveolar macrophages in the PBS control (A), and 6 h (B), 24 h (C), 48 h (D), 72 h (E), and 8 days (F) after the last OVA challenge. Glrx1, green; alveolar macrophage, red; DNA content, blue.

To investigate site-specific Glrx1 temporal expression after OVA-challenge, we performed an immunoblot analysis for Glrx1 protein in BALF and lung tissues. Levels of Glrx1 protein were significantly elevated 6 and 48 h after the last challenge in BALF (p < 0.05) (Fig 1A and S1 Fig, S1 Table) and 48 and 72 h after the last challenge in lung homogenates (p < 0.05) (Fig 1B, S2 Fig, S2 Table). Next, to clarify Glrx1 localization in the lungs, we performed immunohistochemistry for Glrx1 using lung sections and cytospin slides. Compared with the control, we found increased Glrx1 staining intensity in airway epithelial cells (Fig 2) and in alveolar macrophages (Fig 3) after OVA challenge. Glrx1 staining intensity was more evident at 48 and 72 h (Fig 2D and 2E) than 6 h (Fig 2B) after the last OVA challenge in airway epithelial cells. In contrast, Glrx1 levels increased sharply 6 h after the last OVA challenge in alveolar macrophages (Fig 3B), and the expression of Glrx1 persisted for 8 days (Fig 3C–3F).

### *In situ* analysis of S-glutathionylated proteins in lung tissues

**Fig 4:**
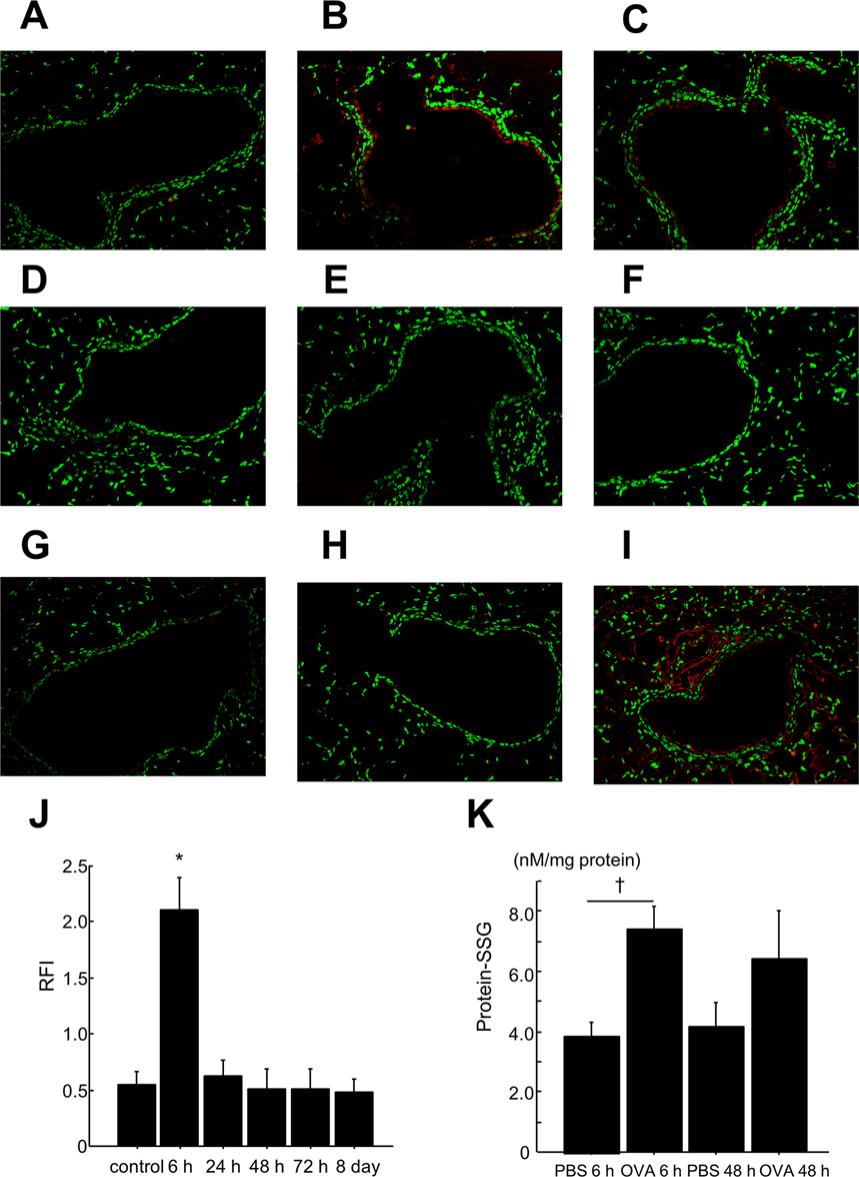
*In situ* analysis of protein—SSG in mouse lung tissue by Glrx1-based cysteine derivatization and biochemical analysis of protein—SSG in lungs after OVA challenges. Representative fluorescent images of lung sections showing protein—SSG reactivity (red) and nuclei (green). Patterns of protein—SSG reactivity in the lungs of PBS control (A) and 6 h (B), 24 h (C), 48 h (D), 72 h (E), and 8 days (F) after the last challenge with OVA. Omission of Glrx1 alone (G) or Glrx1, GSH, GSSG reductase, and NADPH (H) as a negative control. Pretreatment of tissue with diamide and GSH as a positive control (I). Magnification, ×200. (J) Quantitative assessment of the intensity of protein—SSG reactivity in bronchial epithelium. Fluorescent intensity was quantified in bronchial epithelium regions. Mean red fluorescence intensity is divided by mean green fluorescence intensity in the same region to obtain a measure of the relative fluorescence intensity (RFI); data are presented as means ± SEM. *p < 0.05 was considered significant in the comparisons with control mice. (K) The levels of protein—SSG 6 h or 48 h after the last challenge with OVA or PBS were measured by enzyme recycling assay; data are presented as means ± SEM. †p < 0.05 was considered significant in the comparisons with the PBS 6 h group.

To examine whether S-glutathionylation occurs in the lung tissue of OVA-challenged mice, we assessed *in situ* Glrx1-catalyzed cysteine derivatization. The expression of S-glutathionylated proteins in airway epithelial cells 6 h after the last OVA challenge was markedly higher than that in controls (Fig 4A and 4B); however, the expression returned to basal levels thereafter (Fig 4C–4F). Quantitative assessment showed that S-glutathionylated protein expression in airway epithelial cells of the OVA 6 h (final challenge) group was significantly higher compared to controls (p < 0.05) (Fig 4J, S3–S8 Figs, S3 Table).

### Biochemical analysis of protein—SSG in the lungs

We also measured the levels of protein—SSG in lung homogenates by biochemical analysis to confirm the elevation of protein—SSG in this model. We found that protein—SSG levels significantly increased in lung homogenates of the OVA 6 h group, but not of the OVA 48 h group, compared with their respective PBS controls (Fig 4K, S4 Table).

### Total GSH and GSSG in BALF and lung tissues

**Fig 5:**
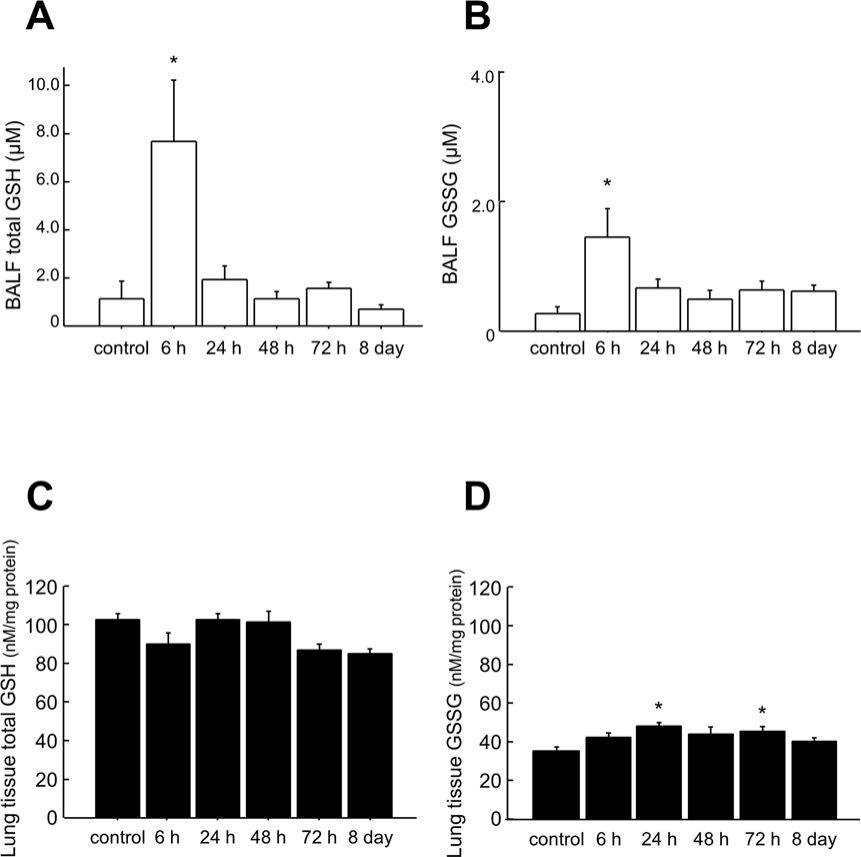
Total GSH and GSSG in the BALF and lung tissue of mice after OVA challenges. Total GSH (A) and GSSG (B) in BALF and total GSH (C) and GSSG (D) in the lungs at the indicated time points were measured using an enzyme recycling assay. Data are presented as means ± SEM. *p < 0.05 was considered significant in the comparisons with control mice.

To assess redox status in this model, we measured total GSH (reduced GSH and GSSG) and GSSG in BALF and lung homogenates. In BALF, concentrations of both total GSH and GSSG increased significantly 6 h after the last challenge compared with control mice (p < 0.05) (Fig 5A and 5B, S5 Table). In lung homogenates, the concentration of total GSH did not change compared with those of control mice (Fig 5C, S6 Table); however, the levels of GSSG significantly increased 24 and 72 h after the last OVA challenge compared with control mice (p < 0.05) (Fig 5D, S6 Table). The ratio of GSSG/total GSH in BALF and lung homogenates did not change compared with control mice (S5 and S6 Tables).

### Cytokine/chemokine profiling in BALF

To investigate the relationship between inflammatory cytokines/chemokines and Glrx1, we measured the levels of 25 cytokines and chemokines in BALF at various time points (S7 Table). The contents of eotaxin-1 and RANTES at 6 and 48 h; IL-4, IL-6, and TNF-α at 6 h; and IL-13 at 6–48 h after the final challenge were significantly higher than the levels in controls in BALF (p < 0.05) (Table 2). Levels of Glrx1 in BALF at all the studied time points were significantly correlated with IL-4, IL-13, eotaxin-1, TNF-α, IL-6, KC, and RANTES. In comparison, the levels of Glrx1 in the lung tissue were significantly correlated only with TGF-β1 (Table 3, S8 Table). Correlation charts between Glrx1 and cytokines/chemokines (IL-4, IL-13, TNF-α, eotaxin-1) are presented in S9 Fig.

**Table 2:** Expression profiles of cytokines and chemokines in BALF.

(pg/ml)	control	OVA 6 h	OVA 24 h	OVA 48 h	OVA 72 h	OVA 8 day
IL-4	2.1±0.0	986.8±212.4*	71.8±46.4	6.5±1.2	4.0±0.4	2.2±0.0
IL-13	0.4±0.1	69.2±16.1*	55.2±19.0*	50.0±12.2*	22.6±10.2	0.3±0.0
TNF-α	0.6±0.1	9.0±1.2*	1.4±0.3	0.9±0.4	0.5±0.1	0.4±0.1
IL-6	0.3±0.0	456.3±139.9*	21.3±12.5	3.7±1.3	0.4±0.0	0.3±0.0
KC	20±2.2	610.3±87.2*	307.0±71.9*	215.8±45.4*	159.8±27.0	107.0±7.9
eotaxin-1	0.6±0.2	25.1±8.4*	16.1±4.9	21.1±4.3*	17.6±3.9	2.6±0.5
RANTES	1.7±0.1	2.5±0.2*	2.1±0.1	2.4±0.2*	2.0±0.1	1.6±0.1
TGF-β1	23.7±3.8	257.8±54.6*	358.7±47.7*	362.7±37.1*	338.4±26.2*	175.7±33.2*

Cytokines and chemokines in BALF were detected at each time point (6 h, 24 h, 48 h, 72 h, and 8 days after the last OVA challenge).

Data are presented as means ± SEM (n = 5).

*p <0.05 compared with control mice.

**Table 3:** Correlations between cytokines/chemokines in BALF and Glrx1 in BALF or lung tissue.

	BALF Glrx1		Lung tissue Glrx1	
IL-4	p<0.01	r = 0.65	p = 0.78	r = 0.05
IL-13	p = 0.01	r = 0.46	p = 0.42	r = 0.15
TNF-α	p<0.01	r = 0.68	p = 0.59	r = 0.10
IL-6	p<0.01	r = 0.62	p = 0.83	r = 0.04
KC	p<0.01	r = 0.61	p = 0.70	r = 0.07
eotaxin-1	p<0.01	r = 0.52	p = 0.18	r = 0.25
RANTES	p<0.01	r = 0.57	p = 0.53	r = 0.12
TGF-β1	p = 0.07	r = 0.33	p = 0.02	r = 0.43

Correlations were assessed by Pearson’s correlation coefficient. All time points are utilized in the correlation analysis.

## Discussion

The present study provides a detailed time-course analysis of the association of levels of Glrx1, protein—SSG, glutathione, and cytokines/chemokines in the lungs of OVA-sensitized BALB/c mice following OVA challenges.

Using biochemical analysis and fluorescent staining of lung tissues, we showed that protein—SSG occurred early after allergen challenges and followed a temporal pattern. However, in a previous study, no clear increases in the overall content of protein—SSG occurred in lung tissues of BALB/c mice after OVA sensitization and challenges [19]. The discrepancy between this result and ours seems can be ascribed to the fact that in that study, protein—SSG was measured only 48 h or 7 days after the last challenge with OVA. It should also be noted that in our model, the levels of protein—SSG in lung tissues and GSH and GSSG in BALF significantly increased at the same time point.

Allergen sensitization and challenges with OVA have been found to immediately induce oxidative stress by increasing intracellular reactive oxygen species in bronchial epithelium [20], and a change in GSH is considered to be one of the oxidative stress markers. Under oxidative stress conditions, protein thiol moieties could be converted to protein—SSG mixed disulfide adducts by various biochemical mechanisms, namely, via (1) thiol-disulfide exchange, (2) sulfenic acid intermediates, (3) sulfenamide intermediates, (4) thiyl radical intermediates, (5) thiosulfinate intermediates, or (6) S-nitrosyl intermediates (10). Taken together, our results suggest that allergen sensitization and challenges immediately lead to protein—SSG formation under oxidative stress by such mechanisms, and the protein—SSG returns to baseline levels thereafter. Since S-glutathionylation protects the targeted protein thiol groups from further irreversible oxidations, increased levels of protein—SSG would be a physiological defense mechanism in this model. Notably, Glrx1 protein levels in induced sputum were reported to be significantly higher, while protein—SSG levels were significantly lower, in asthmatic individuals compared with healthy controls [21].

Glrx1 specifically catalyzes de-glutathionylation under physiological conditions by removing conjugated glutathione from proteins [22]. Since Glrx1 was induced in the bronchial epithelium and alveolar macrophages in this study, which is consistent with previous reports [11, 23], decreased protein—SSG observed at later time points may have resulted from Glrx1-catalyzed de-glutathionylation. However, Glrx1 increased sharply at 6 h in alveolar macrophages, while it increased only slightly in the bronchial epithelium at that time point. Moreover, we observed a discrepancy between Glrx1 levels in BALF and macrophage numbers considering that alveolar macrophages were the main source of Glrx1 in BALF. This discrepancy was also found in another report [23]. Although it remains to be unraveled whether alveolar macrophages and/or bronchial epithelial cells actually release Glrx1, we presume that the increase in Glrx1 in alveolar macrophages and bronchial epithelial cells might be related to the increase in the levels of Glrx1 in BALF.

Reversible protein—SSG (S-glutathionylation and de-glutathionylation) modulates functions in proteins such as actin [24, 25], Ras [26], protein tyrosine phosphatases [27–29], and NF-κB [4, 5]. NF-κB is an important transcription factor that regulates allergic airway inflammation [6–8]. S-glutathionylation of the p50 and p65 subunits of NF-κB or the inhibitory κB kinase (IKK) β subunit inhibits NF-κB binding to DNA and consequent inflammatory gene transcription [4, 5, 16, 30]. In contrast, the de-glutathionylation of IKK by the upregulation of Glrx1 was found to prolong NF-κB activation and increase the levels of proinflammatory mediators [31, 32]. Furthermore, genetic ablation of Glrx1 decreases eosinophilic inflammation, as well as the expression of pro-inflammatory NF-κB-related mediators like KC and CCL20, and enhances the resolution of airway hyperresponsiveness and mucus metaplasia in mice with allergic airway inflammation [19]. Therefore, decreasing Glrx1 and enhancing protein—SSG could resolve inflammation/cytokine production in allergic airway inflammation. In our study, the increases in Glrx1 in BALF corresponded with the timing of the increases in Th2/NF-κB-related cytokines and chemokines, and the increased levels of Glrx1 in BALF and lung tissues lasted throughout the period of eosinophil infiltration in the airway, along with inflammatory cytokines/chemokines in BALF. These findings suggest that Glrx1 might be involved in allergic airway inflammation through the regulation of the NF-κB pathway by modulating protein—SSG redox status.

The present study showed dynamic changes in Glrx1 and protein—SSG redox status and cytokines/chemokines in the lungs of a murine model of allergic airway inflammation. Glrx1 and protein—SSG redox status may play important roles in the development of bronchial asthma and regulating those status could be a potentially clinical intervention to attenuate allergic airway inflammation.

## Supporting Information

S1 Fig Expression of Glrx1 in the BALF of mice after OVA challenge. BALF from OVA-challenged mice was analyzed by western blot analysis for Glrx1 expression at the indicated time points (6, 24, 48, and 72 h, and 8 days after the last challenge with OVA).
(TIF)

S2 Fig Expression of Glrx1 in the lung tissues of mice after OVA challenge. Lung homogenates from OVA-challenged mice were analyzed by western blot analysis for Glrx1 expression at the indicated time points (6, 24, 48, and 72 h, and 8 days after the last challenge with OVA). Actin was used as a loading control for lung homogenates.
(TIF)

S3 Fig *In situ* analysis of protein—SSG in mouse lung tissues (control) by Glrx1-based cysteine derivatization. Fluorescent images of lung sections showing protein—SSG reactivity (red) and nuclei (green). Patterns of protein—SSG reactivity in the lungs of mice treated with PBS (control). Magnification, ×200.
(TIF)

S4 Fig *In situ* analysis of protein—SSG in mouse lung tissues (OVA 6 h group) by Glrx1-based cysteine derivatization. Fluorescent images of lung sections showing protein—SSG reactivity (red) and nuclei (green). Patterns of protein—SSG reactivity in the lungs of mice 6 h after the last challenge with OVA. Magnification, ×200.
(TIF)

S5 Fig *In situ* analysis of protein—SSG in mouse lung tissues (OVA 24 h group) by Glrx1-based cysteine derivatization. Fluorescent images of lung sections showing protein—SSG reactivity (red) and nuclei (green). Patterns of protein—SSG reactivity in the lungs of mice 24 h after the last challenge with OVA. Magnification, ×200.
(TIF)

S6 Fig *In situ* analysis of protein—SSG in mouse lung tissues (OVA 48 h group) by Glrx1-based cysteine derivatization. Fluorescent images of lung sections showing protein—SSG reactivity (red) and nuclei (green). Patterns of protein—SSG reactivity in the lungs of mice 48 h after the last challenge with OVA. Magnification, ×200.
(TIF)

S7 Fig *In situ* analysis of protein—SSG in mouse lung tissues (OVA 72 h group) by Glrx1-based cysteine derivatization. Fluorescent images of lung sections showing protein—SSG reactivity (red) and nuclei (green). Patterns of protein—SSG reactivity in the lungs of mice 72 h after the last challenge with OVA. Magnification, ×200.
(TIF)

S8 Fig *In situ* analysis of protein—SSG in mouse lung tissues (OVA 8 days group) by Glrx1-based cysteine derivatization. Fluorescent images of lung sections showing protein—SSG reactivity (red) and nuclei (green). Patterns of protein—SSG reactivity in the lungs of mice 8 days after the last challenge with OVA. Magnification, ×200.
(TIF)

S9 Fig Correlation charts between Glrx1 and cytokines/chemokines per time point in the BALF of mice after OVA challenge. Correlations charts between Glrx1 and IL-4, IL-13, TNF-α, or eotaxin-1 colored different time points of dots in different colors.
(TIF)

S1 Table Expression of Glrx1 in the BALF of mice after OVA challenge. BALF from OVA-challenged mice were analyzed by western blot analysis for Glrx1 expression at the indicated time points (6, 24, 48, and 72 h, and 8 days after the last challenge with OVA). On each blot, the detected Glrx1 band intensity was divided by that of a representative standard, which was used on every blot.
(XLSX)

S2 Table Expression of Glrx1 in the lung tissues of mice after OVA challenge. Lung homogenates from OVA-challenged mice were analyzed by western blot analysis for Glrx1 expression at the indicated time points (6, 24, 48, and 72 h, and 8 days after the last challenge with OVA). Actin was used as a loading control for lung homogenates. On each blot, the detected Glrx1 band intensity was divided by the actin band intensity as a loading control and then divided by the value of the representative standard (Glrx1/actin), which was used on every blot.
(XLSX)

S3 Table *In situ* analysis of protein—SSG in mouse lung tissues by Glrx1-based cysteine derivatization. Quantitative assessment of the intensity of protein—SSG reactivity in the bronchial epithelium was conducted by evaluating the mean red fluorescence intensity (protein—SSG) in each region of interest and dividing this by the mean green fluorescence intensity (DNA content) present in the same region, thereby obtaining the relative fluorescence intensity (RFI) of protein—SSG.
(XLSX)

S4 Table Biochemical analysis of protein—SSG in the lungs after OVA challenge. protein—SSG in lung tissues was determined using a glutathione/glutathione reductase/NADPH/5,5ʹ-dithiobis-(2-nitrobenzoic acid) (DTNB) recycling assay.
(XLSX)

S5 Table Total GSH, GSSG, and the ratio of GSSG/total GSH in the BALF of mice after OVA challenge. Total GSH and GSSG were measured in BALF using the DTNB recycling assay.
(XLSX)

S6 Table Total GSH, GSSG, and the ratio of GSSG/total GSH in the lung tissues of mice after OVA challenge. Total GSH and GSSG were measured in deproteinated lung homogenates using the DTNB recycling assay.
(XLSX)

S7 Table Expression profile of 25 cytokines and chemokines in BALF. Cytokine and chemokine concentrations in the BALF of mice 6, 24, 48, or 72 h or 8 days after the last challenge with OVA were measured using a MILLIPLEX Mouse Cytokine/Chemokine kit. Data are presented as means ± SEM (n = 5). * p < 0.05 was considered significant in the comparisons with control mice. ND: not detectable.
(XLSX)

S8 Table Correlations of cytokines/chemokines and Glrx1 per time point in the BALF and lung tissues of mice after OVA challenge. Correlations were assessed by Pearson’s correlation coefficient.
(XLSX)
