# Supplementary figures and images for: Temporal Changes in Glutaredoxin 1 and Protein S-Glutathionylation in Allergic Airway Inflammation
Source: PLoS One. 2015 Apr 13;10(4):e0122986. doi: 10.1371/journal.pone.0122986 (PMC4395207; doi:10.1371/journal.pone.0122986)

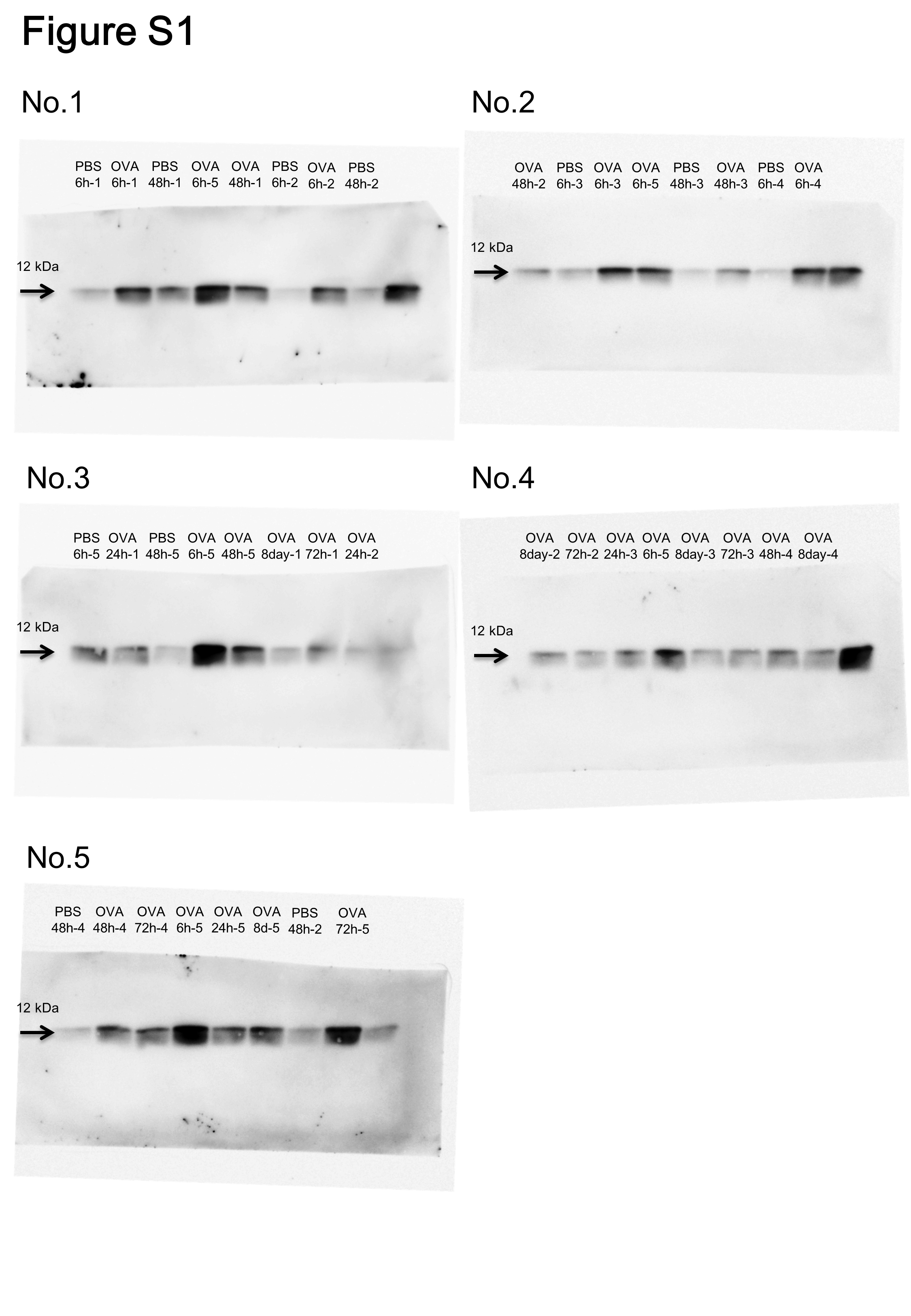

Supplement: S1 Fig — BALF from OVA-challenged mice was analyzed by western blot analysis for Glrx1 expression at the indicated time points (6, 24, 48, and 72 h, and 8 days after the last challenge with OVA). (TIF) [file pone.0122986.s001.tif]

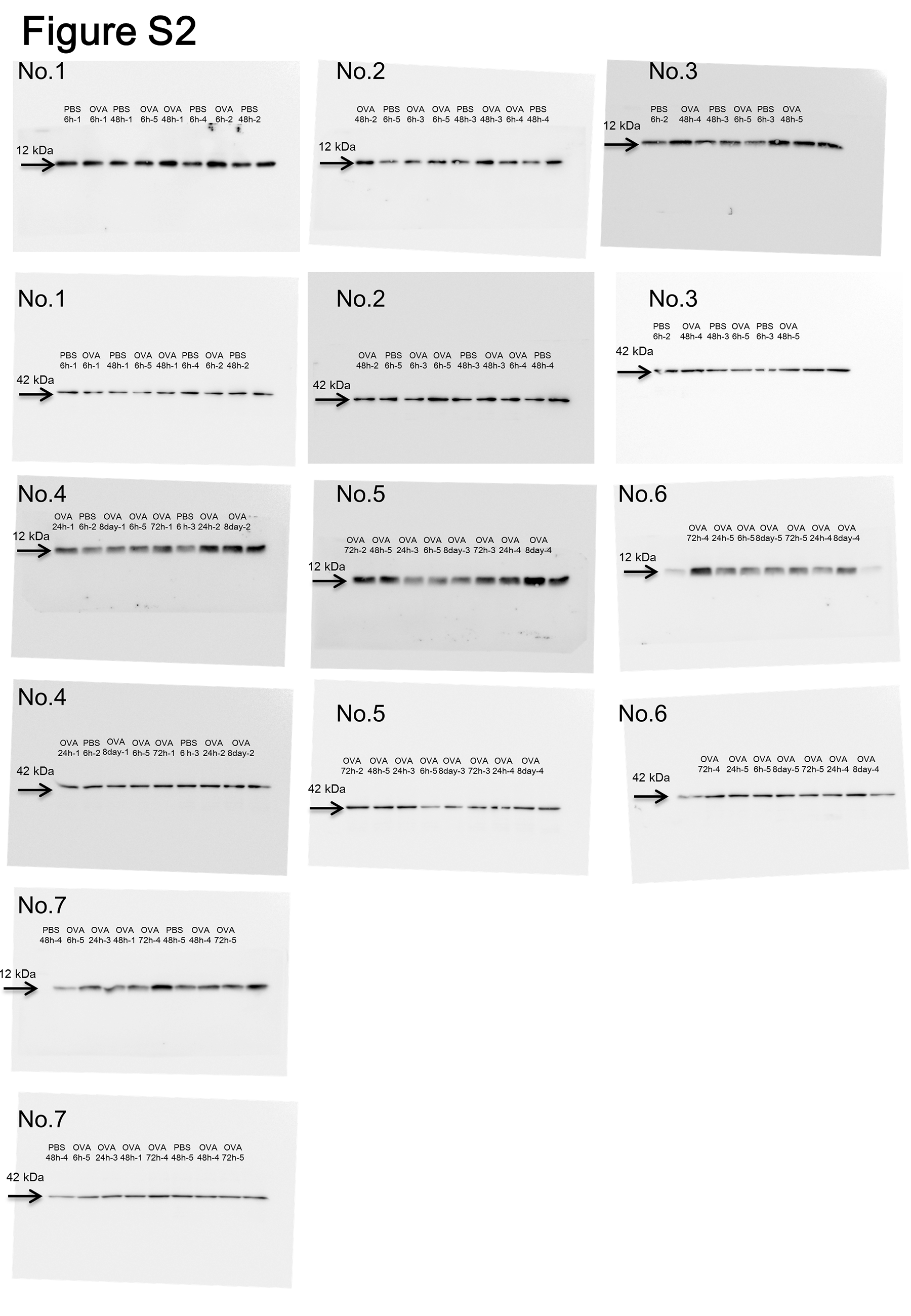

Supplement: S2 Fig — Lung homogenates from OVA-challenged mice were analyzed by western blot analysis for Glrx1 expression at the indicated time points (6, 24, 48, and 72 h, and 8 days after the last challenge with OVA). Actin was used as a loading control for lung homogenates. (TIF) [file pone.0122986.s002.tif]

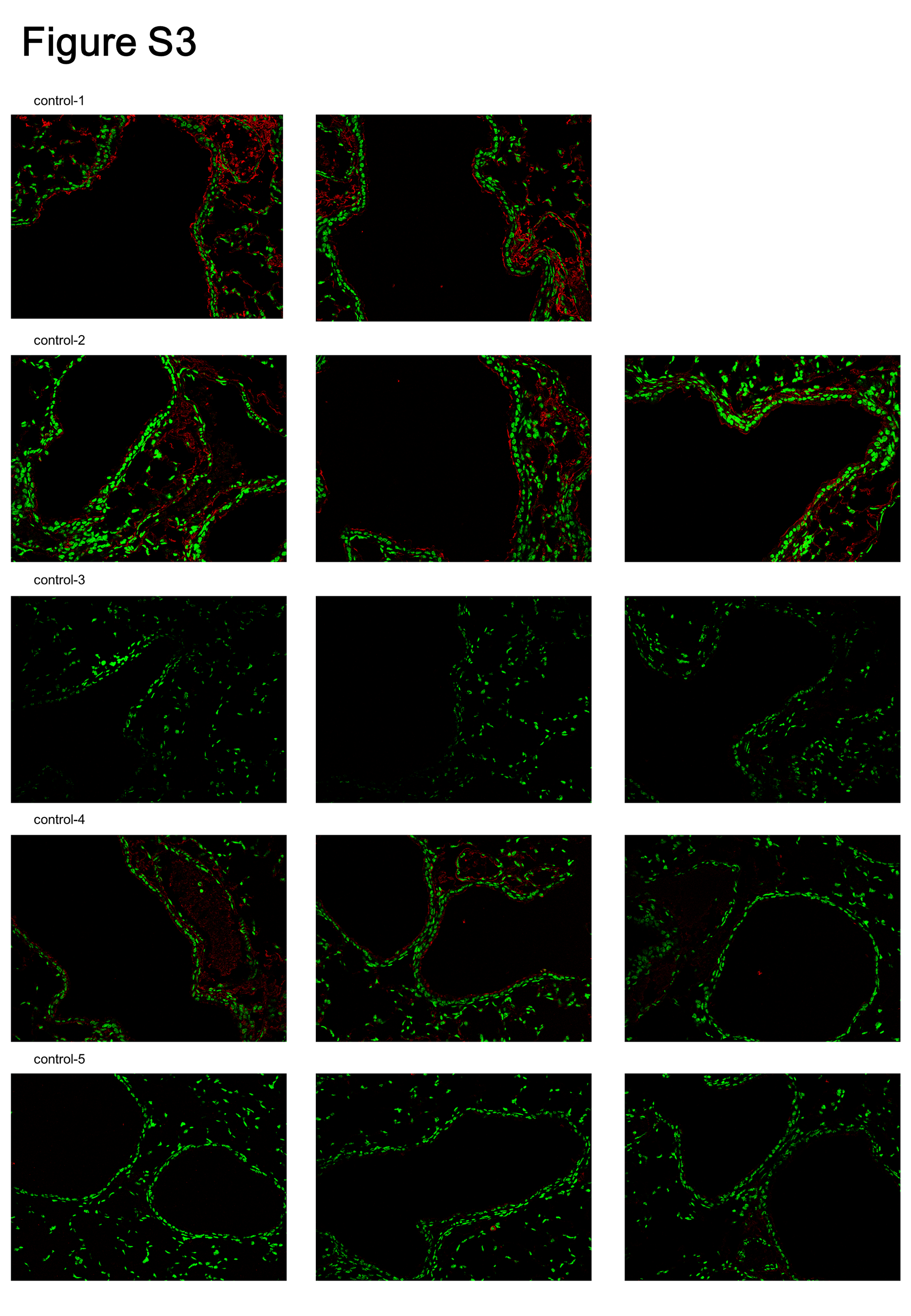

Supplement: S3 Fig — Fluorescent images of lung sections showing protein—SSG reactivity (red) and nuclei (green). Patterns of protein—SSG reactivity in the lungs of mice treated with PBS (control). Magnification, ×200. (TIF) [file pone.0122986.s003.tif]

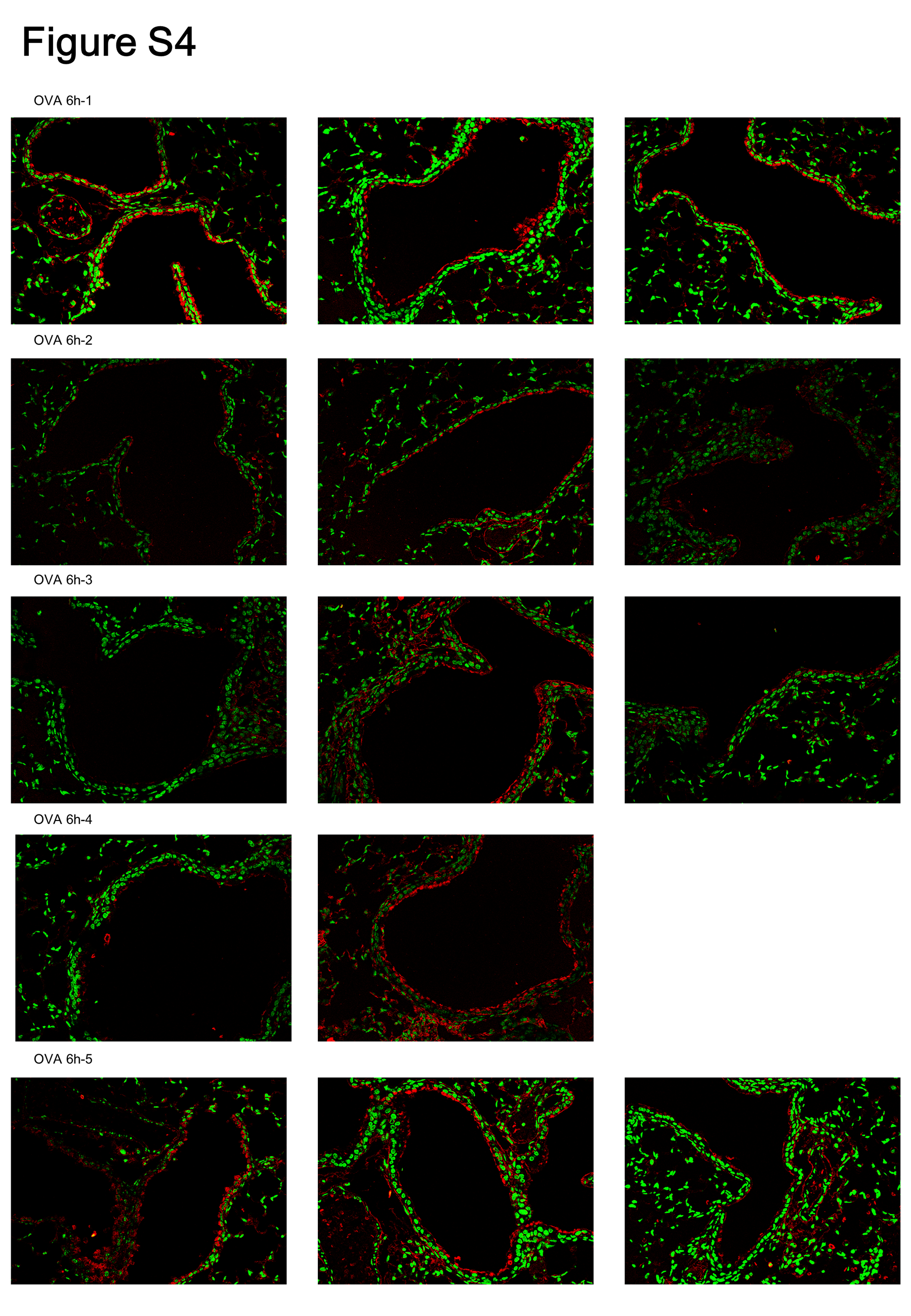

Supplement: S4 Fig — Fluorescent images of lung sections showing protein—SSG reactivity (red) and nuclei (green). Patterns of protein—SSG reactivity in the lungs of mice 6 h after the last challenge with OVA. Magnification, ×200. (TIF) [file pone.0122986.s004.tif]

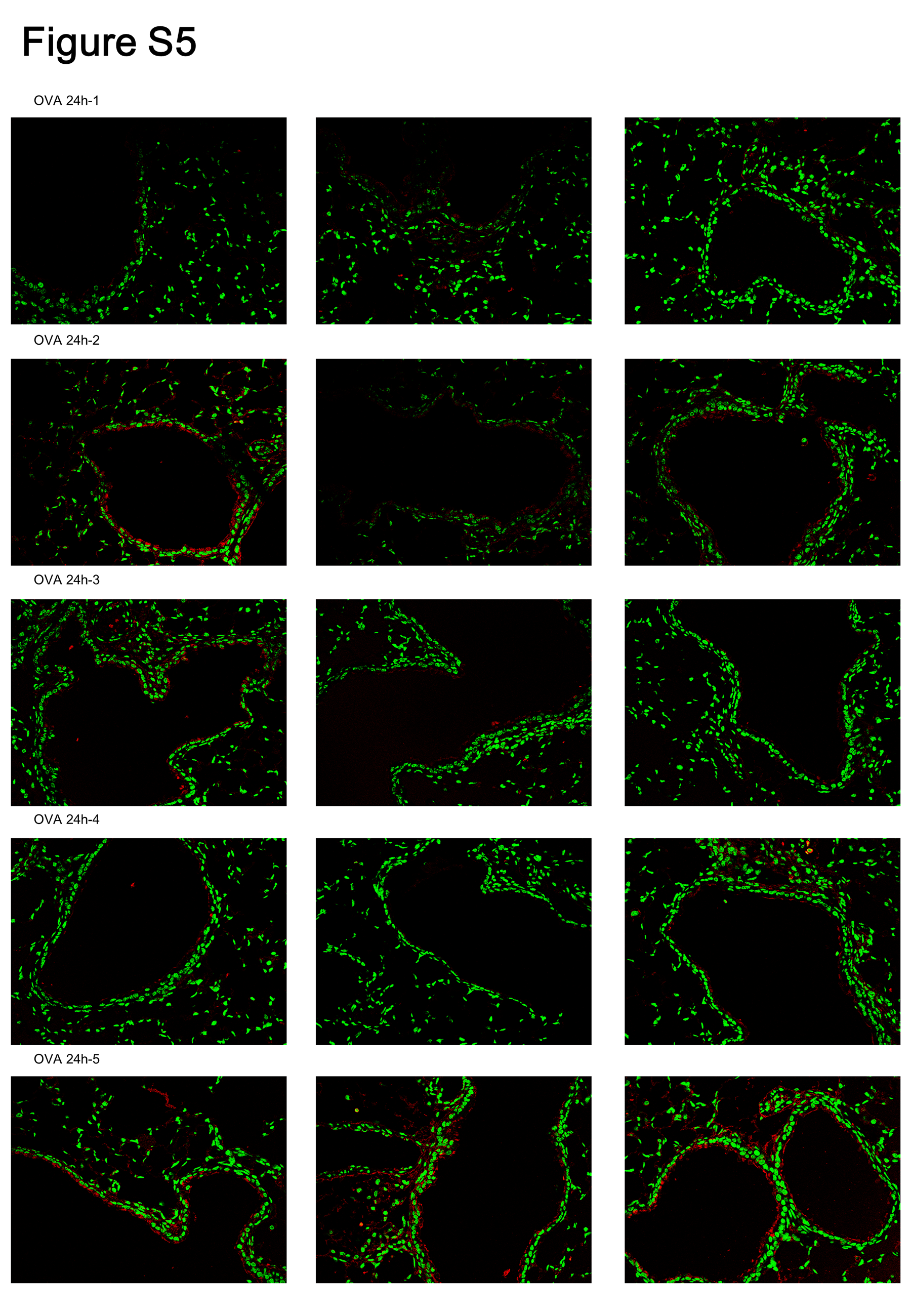

Supplement: S5 Fig — Fluorescent images of lung sections showing protein—SSG reactivity (red) and nuclei (green). Patterns of protein—SSG reactivity in the lungs of mice 24 h after the last challenge with OVA. Magnification, ×200. (TIF) [file pone.0122986.s005.tif]

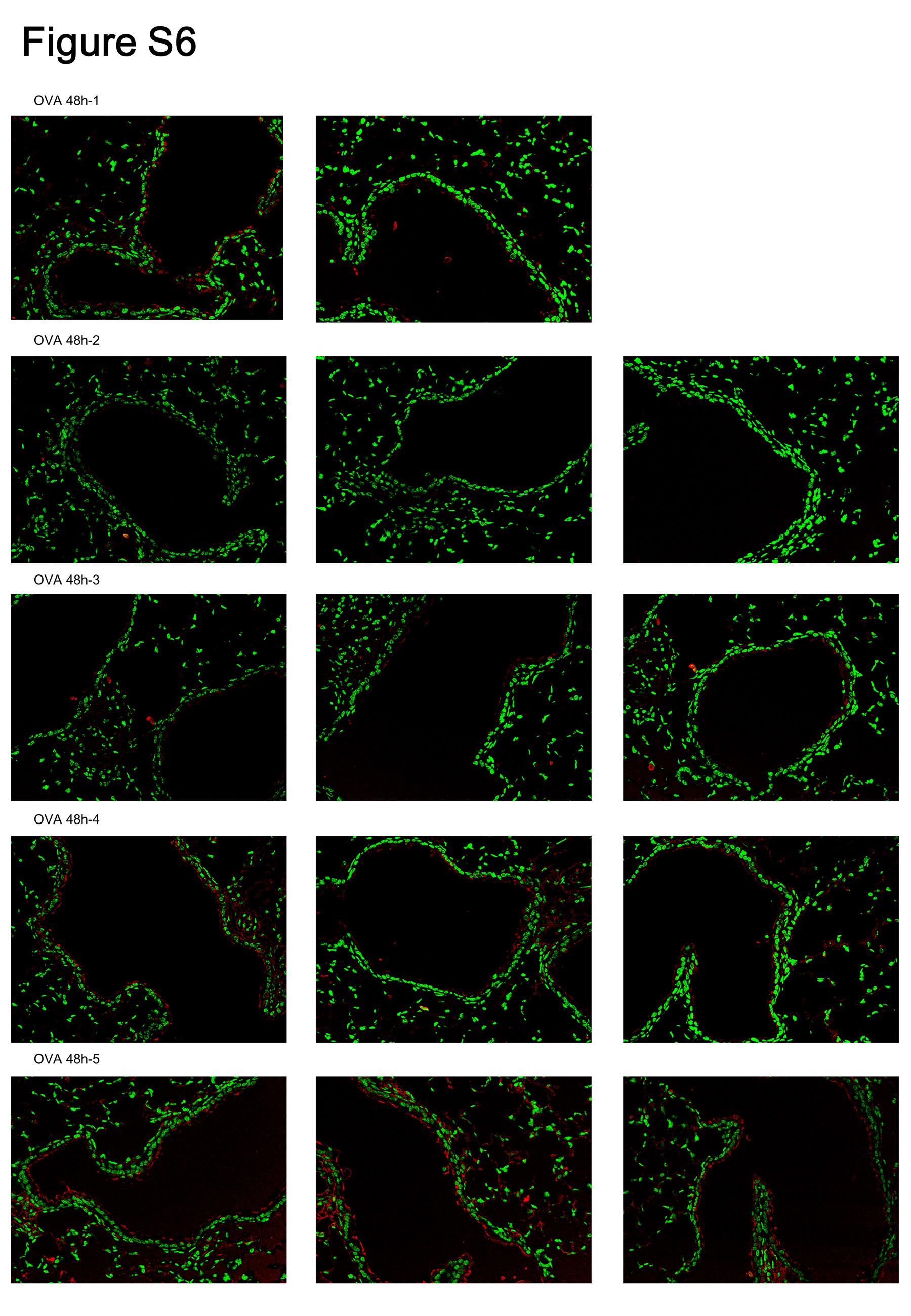

Supplement: S6 Fig — Fluorescent images of lung sections showing protein—SSG reactivity (red) and nuclei (green). Patterns of protein—SSG reactivity in the lungs of mice 48 h after the last challenge with OVA. Magnification, ×200. (TIF) [file pone.0122986.s006.tif]

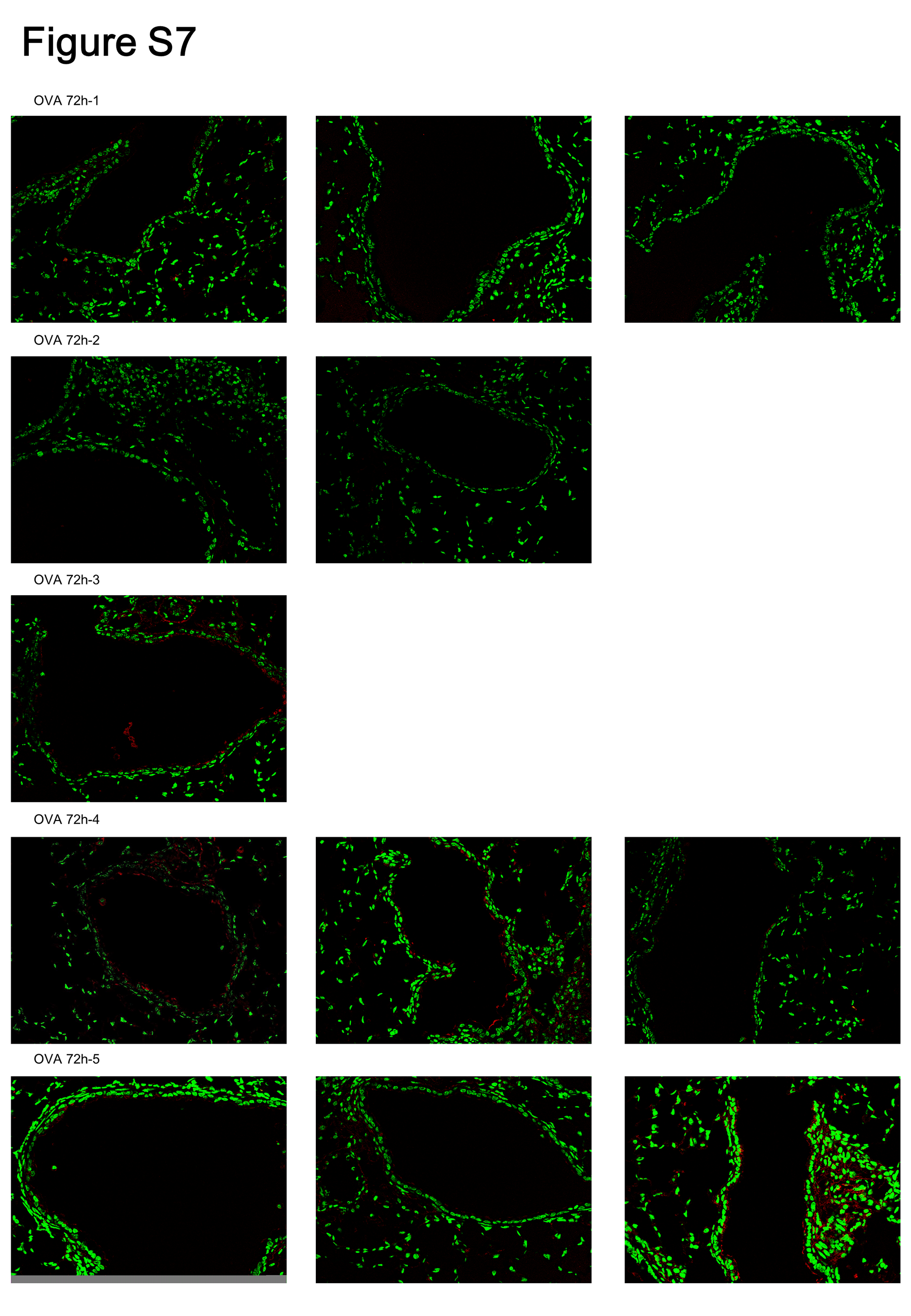

Supplement: S7 Fig — Fluorescent images of lung sections showing protein—SSG reactivity (red) and nuclei (green). Patterns of protein—SSG reactivity in the lungs of mice 72 h after the last challenge with OVA. Magnification, ×200. (TIF) [file pone.0122986.s007.tif]

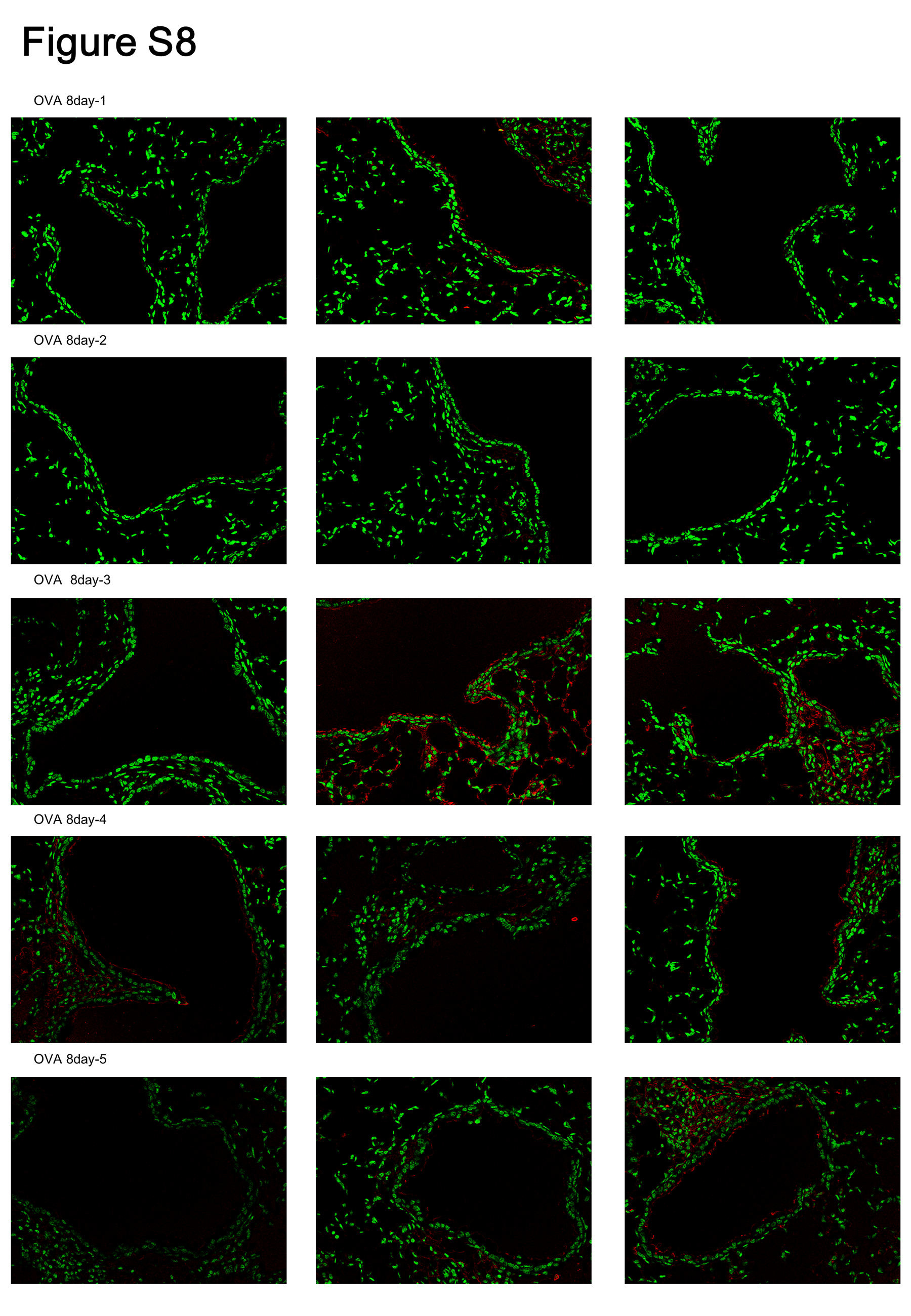

Supplement: S8 Fig — Fluorescent images of lung sections showing protein—SSG reactivity (red) and nuclei (green). Patterns of protein—SSG reactivity in the lungs of mice 8 days after the last challenge with OVA. Magnification, ×200. (TIF) [file pone.0122986.s008.tif]

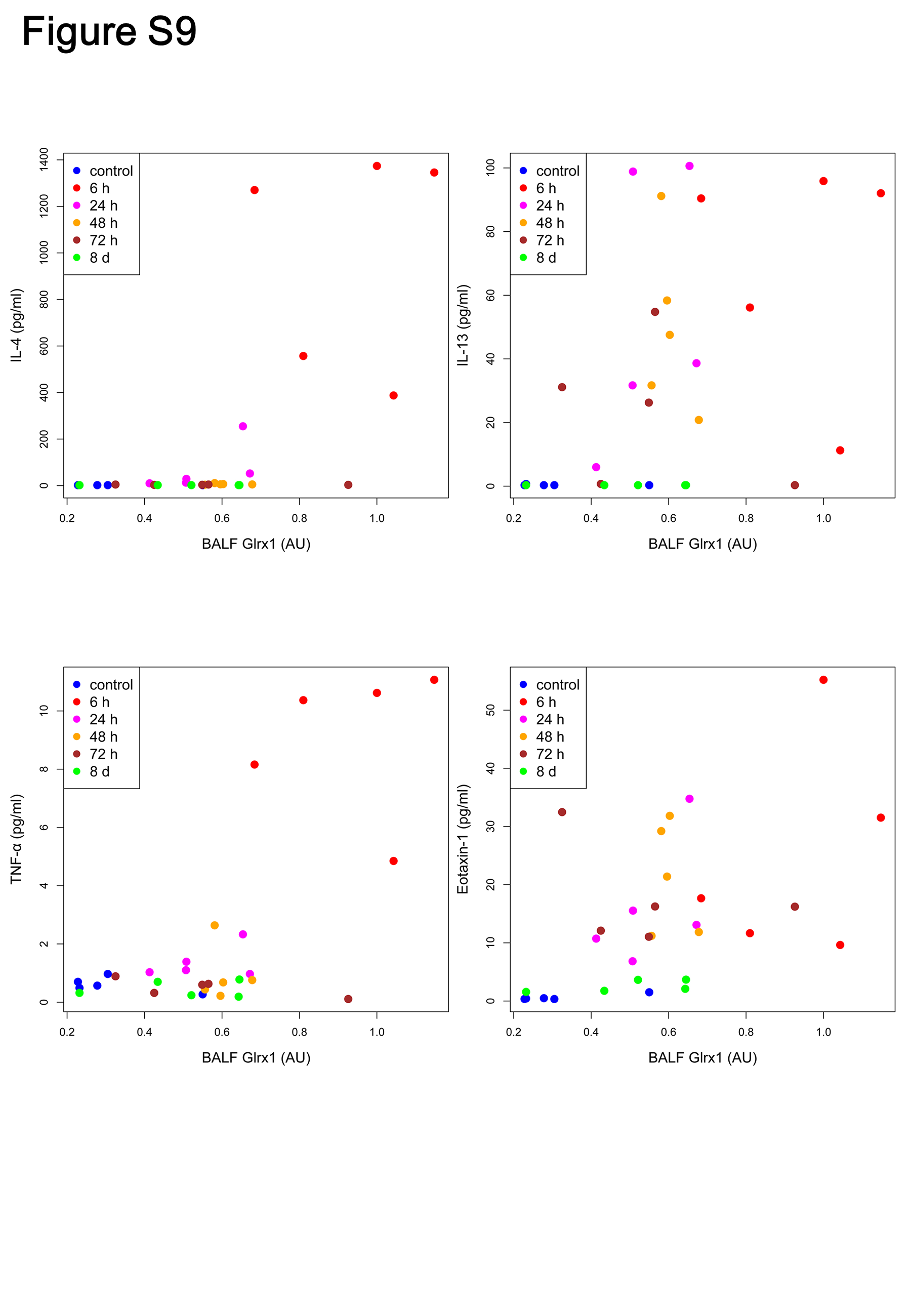

Supplement: S9 Fig — Correlations charts between Glrx1 and IL-4, IL-13, TNF-α, or eotaxin-1 colored different time points of dots in different colors. (TIF) [file pone.0122986.s009.tif]
